# Stratified Psychosocial Care in Assisted Reproductive Technologies: A Narrative Review and Conceptual Framework Across the IVF Pathway

**DOI:** 10.3390/jcm15135117

**Published:** 2026-07-01

**Authors:** Giuseppe Marano, Giulio Carriero, Elena Lucia Valle, Caterina Brisi, Gianandrea Traversi, Osvaldo Mazza, Claudia d’Abate, Rosanna Esposito, Gabriele Sani, Marianna Mazza

**Affiliations:** 1 Department of Neuroscience, Head-Neck and Chest, Section of Psychiatry, Fondazione Policlinico, Universitario Agostino Gemelli IRCCS, Largo Agostino Gemelli 8, 00168 Rome, Italyelenalucia.v@gmail.com (E.L.V.);; 2 Department of Neuroscience, Section of Psychiatry, Università Cattolica del Sacro Cuore, 00168 Rome, Italy; 3 Unit of Medical Genetics, Department of Laboratory Medicine, Ospedale Isola Tiberina-Gemelli Isola, 00186 Rome, Italy; gianandrea.traversi@gmail.com; 4 Spine Surgery Department, Bambino Gesù Children’s Hospital IRCCS, 00168 Rome, Italy; osvaldo.mazza1973@hotmail.it; 5 Department of Gynecology, San Carlo Nancy Hospital, 00165 Rome, Italy; claudiadabate94@gmail.com; 6 Unit of Gynecology and Obstetrics, A.O.R.N. Antonio Cardarelli Hospital, 80131 Naples, Italy; dr.rosannaesposito@gmail.com

**Keywords:** assisted reproductive technologies, IVF, infertility-related distress, precision psychosocial care, screening, mental health

## Abstract

Assisted reproductive technologies (ART), particularly in vitro fertilization (IVF), are characterized by substantial emotional burden, uncertainty, and repeated decision-making under probabilistic conditions. Although psychological distress is common among individuals and couples undergoing ART, psychosocial care remains inconsistently integrated into fertility pathways and is rarely tailored to individual needs. This narrative review synthesizes evidence concerning infertility-related distress, anxiety and depressive symptoms, couple functioning, treatment discontinuation, psychosocial screening, and psychological interventions across the IVF trajectory. Key phases of treatment, including pretreatment assessment, ovarian stimulation, embryo transfer, the waiting period, cycle outcome, and decisions regarding repetition or discontinuation, are described, and phase-specific psychological vulnerabilities are identified. On the basis of the available literature, we propose a conceptual stepped-care framework ranging from universal psychoeducation and routine monitoring to structured psychological interventions, couple-based care, and specialist psychiatric management. Practical screening thresholds, referral criteria, reassessment intervals, and a safety pathway for suicidal ideation are outlined. Particular attention is given to male-factor infertility, strategies for engaging male partners, and the ethical and data-governance requirements of digital mental health tools. Current evidence supports psychosocial interventions for improving emotional well-being and quality of life and suggests a potential role in treatment continuation, but their effects on pregnancy and live-birth outcomes remain uncertain. The proposed framework should therefore be regarded as a clinically informed conceptual model rather than a validated prediction algorithm. Prospective studies are required to assess its feasibility, predictive performance, cost-effectiveness, and impact on patient-centered outcomes.

## 1. Introduction

Infertility is defined as a disease of the male or female reproductive system characterized by the failure to achieve pregnancy after 12 months or more of regular unprotected sexual intercourse, while also encompassing impairments in an individual’s capacity to reproduce either alone or with a partner [1,2]. Available global estimates indicate that infertility affects a substantial proportion of the reproductive-age population, although reported prevalence varies according to the population examined, the definition applied, and the estimation method [3]. This public-health burden has increased attention to equitable access to medically assisted reproduction (MAR), including intrauterine or other forms of artificial insemination and assisted reproductive technologies (ART) such as in vitro fertilization (IVF), intracytoplasmic sperm injection, and frozen embryo transfer.

However, ART pathways are frequently associated with substantial emotional distress arising from uncertainty regarding treatment outcomes, the physical and organizational burden of procedures, conflicts between treatment and occupational responsibilities, financial costs, insufficient social support, and repeated exposure to reproductive loss or failure. Psychological distress may compromise well-being, quality of life, couple functioning, and continuity of care [4]. Its direct relationship with reproductive outcomes, however, remains uncertain. Biological hypotheses involving the hypothalamic–pituitary–adrenal and hypothalamic-pituitary-ovarian axes, inflammatory signaling, and oxidative stress have been proposed, but clinical findings have been inconsistent [5,6]. Notably, a meta-analysis of prospective psychosocial studies found no significant association between pretreatment emotional distress and pregnancy achievement after a cycle of assisted reproductive technology [7]. Subsequent reviews have similarly emphasized substantial heterogeneity and confounding by maternal age, infertility diagnosis, previous treatment exposure, ART protocol, and baseline reproductive prognosis. Psychological distress should therefore not be presented as an established direct cause of IVF failure. The strongest justification for psychosocial care lies in its potential to improve emotional well-being, quality of life, informed decision-making, couple adjustment, and treatment continuity. Distress may also indirectly influence cumulative treatment opportunity when it contributes to postponement or premature discontinuation of otherwise indicated care [8].

Despite high prevalence of anxiety, depression, and stress symptoms among infertile patients [4], and despite evidence that psychological interventions are effective in reducing psychosocial burden and improving well-being in patients experiencing infertility-related distress, the integration of such interventions into infertility treatment protocols remains limited to date, and even if they are provided, patients frequently do not access them due to insufficient information or fear of stigmatization: a 2016 study conducted at the University of California found that only 26.7% of women and 24.1% of men reported that the clinic where they sought fertility treatment provided them with information about professional mental health services (MHS), while only 21% of women and 11.3% of men reported having received MHS to address personal or relationship issues related to their difficulty conceiving at some point during the study period [9]. Moreover, there remains a significant shortage of mental health professionals specialized in providing psychological care to patients undergoing assisted reproductive technology treatment. Another issue is often represented by patients’ difficulty in accessing traditional face-to-face mental health care. A 2023 systematic review highlighted that psychological and cognitive interventions targeting emotional distress in patients undergoing assisted reproductive technologies are associated with statistically significant improvements in psychological outcomes, with modest but positive effects also observed in fertilization outcomes. However, the magnitude of these effects remains limited, potentially due to barriers in access to and continuity of care, the persistence of social stigma, and the limited specificity of existing therapeutic approaches in addressing the psychological burden uniquely associated with infertility and ART treatment pathways [10,11]. Accordingly, while these interventions exhibit clinical utility, there is a clear need for the development and implementation of novel psychological approaches to infertility-related distress. Such interventions should be more accessible, adaptable to patients’ needs, and specifically tailored to the psychosocial challenges inherent to infertility and its treatment. Importantly, they should be systematically and routinely integrated into standard care protocols for individuals undergoing fertility treatment.

This review distinguishes three clinically related but evidentially separate outcomes: improvement in psychological symptoms and quality of life; support for adherence and treatment continuation; and possible effects on pregnancy or live-birth rates. Evidence is strongest for the first domain, clinically plausible but less definitive for the second, and currently inconsistent for the third. The stratified-care framework proposed here should consequently be interpreted as a conceptual clinical model requiring prospective validation rather than as an established predictive algorithm.

## 2. Materials and Methods

This article was designed as a narrative review with a clinically oriented conceptual synthesis. The purpose of the literature search was to identify representative empirical studies, systematic reviews, meta-analyses, professional recommendations, and validated assessment instruments relevant to psychosocial care during infertility treatment, rather than to conduct an exhaustive systematic review or formal evidence-grading exercise.

PubMed/MEDLINE, Scopus, Web of Science, and PsycINFO were consulted for English-language publications issued between January 2000 and May 2026. Search concepts included combinations of terms relating to infertility and assisted reproduction, such as “infertility,” “assisted reproductive technology,” “in vitro fertilization,” and “intracytoplasmic sperm injection,” with terms relating to “psychological distress,” “anxiety,” “depression,” “quality of life,” “couple functioning,” “dyadic coping,” “screening,” “SCREENIVF,” “psychological intervention,” “cognitive behavioral therapy,” “digital mental health,” “treatment discontinuation,” and “dropout.” Reference lists of relevant reviews, guidelines, and frequently cited studies were also examined to identify additional publications.

Priority was given to studies involving adults undergoing infertility assessment or treatment and to publications addressing validated screening instruments, psychological or psychiatric interventions, couple-based care, digital support, treatment continuation, or integrated service models. Case reports, non-clinical animal studies, conference abstracts, editorials, and publications without direct relevance to psychosocial care in assisted reproduction were generally not considered.

The literature was organized narratively according to the following clinically relevant themes: psychological burden associated with infertility; changes in distress across the IVF trajectory; psychosocial screening and risk indicators; stepped and stratified interventions; couple and male-factor considerations; treatment discontinuation; digital care; and multidisciplinary implementation. Because the review did not use an exhaustive protocol, formal study-selection counts, risk-of-bias assessment, or PRISMA reporting were not applied. The resulting synthesis is intended to support conceptual and clinical integration rather than provide pooled estimates of intervention efficacy.

## 3. Psychological Burden in ART: Beyond Anxiety and Depression

Infertility represents a significant risk factor for the development of psychiatric conditions, with anxiety and depressive symptoms being markedly more prevalent among infertile individuals compared with the general population. The prevalence of these disorders is also higher among infertile individuals undergoing ART compared with those who do not pursue such treatment, reaching rates of 48% in women and 28.4% in men for anxiety, and 35.6% in women and 18.6% in men for depression [4]. Suicidal thoughts and behaviors represent an important but insufficiently characterized concern in infertility care. One exploratory single-center cross-sectional study involving 100 women undergoing infertility treatment reported suicidal risk or ideation in approximately 25% of participants using the Mini International Neuropsychiatric Interview. Given the limited sample size, single-center design, sex-specific population, and exploratory nature of the investigation, this figure should not be interpreted as a population prevalence estimate or directly compared with general-population rates. Nevertheless, the finding supports the need for routine assessment of self-harm and suicide risk when patients report severe depression, hopelessness, treatment exhaustion, reproductive loss, or marked functional deterioration [12]. The psychological burden experienced by patients with infertility undergoing ART cannot be limited to anxiety and depressive symptoms alone, but also involves a range of condition-specific psychological and psychosocial factors that contribute to the worsening of psychological distress.

### 3.1. Infertility-Related Distress

According to the transactional model of stress, the stress process involves three main components: the presence of an environmental stressor, the individual cognitive appraisal of such stressors, and the subsequent affective, behavioral, and biological responses [13]. Emotional distress is a state of psychological suffering that may arise as a direct consequence of these stressors or of the stress process itself; however, unlike stress, it is not necessarily characterized by a direct or univocal relationship with external events. Infertility-related distress has been defined as the emotional strain associated with the inability to conceive or achieve parenthood, and it is essential to distinguish it from stress, as it is not solely associated with the clinical diagnosis of infertility or a specific medical procedure, but extends to a range of existential, social, and relational experiences related to this condition. Patel et al. identified several major theoretical frameworks contributing to the development of infertility-related distress, highlighting the multidimensional nature of psychological suffering in infertility [14]. Among these, grief and bereavement models describe infertility as a form of chronic and cyclical loss experienced by both partners. Such loss involves multiple domains, including the loss of reproductive and parental identity, life goals, self-esteem, bodily control, social role, family continuity, and psychological well-being. Unlike conventional bereavement processes, infertility-related grief often becomes repetitive and cyclical, re-emerging with each menstrual cycle, failed treatment attempt, or pregnancy loss, and may persist until parenthood is achieved or the couple renounces the desire for a child. Patel et al. described this phenomenon as the “28-day grief cycle of infertility,” emphasizing the recurrent nature of emotional suffering associated with infertility treatments. Identity-based approaches suggest that infertility may threaten core aspects of self-concept and identity coherence, particularly in sociocultural contexts in which fertility and parenthood are strongly associated with femininity, masculinity, adulthood, or personal fulfillment. Children may be perceived as an extension of the self and of family lineage, such that infertility can cause reduced self-worth and self-image, leading to narcissistic injury. Stress and coping theories instead consider infertility as a chronic stressor characterized by uncertainty, unpredictability, uncontrollability, and repeated exposure to emotionally salient events. According to these models, psychological outcomes are shaped not only by the infertility condition itself, but also by individual cognitive appraisals, coping resources, emotional regulation strategies, and illness representations. Recent multidimensional models, such as the infertility-related stress model (I-STRESS), further emphasize the interaction between infertility-related stressors, coping strategies, psychological health conditions, and dyadic adjustment in determining patients’ emotional outcomes. In this regard, social concern, avoidant coping strategies, prolonged infertility duration, and repeated treatment cycles appear to be associated with greater psychological vulnerability and poorer adjustment during ART [15]. At the same time, social construction perspectives emphasize the sociocultural meanings attributed to infertility and involuntary childlessness. In many cultural contexts, infertility may be associated with stigma, shame, social exclusion, and perceived social deviance, leading individuals to internalize feelings of guilt, inadequacy, or defectiveness. From a relational perspective, infertility may disrupt expected developmental transitions and alter communication patterns, intimacy, sexual functioning, emotional boundaries, and dyadic coping processes. Phase or stage theories further describe infertility-related distress as a dynamic process evolving across different stages of the reproductive journey, from the initial realization of fertility difficulties and diagnostic evaluation to immersion in treatment procedures, repeated failures, and eventual resolution or adaptation. This perspective highlights how emotional needs and psychological vulnerabilities may substantially vary across different phases of the ART trajectory. Recent evidence also highlights the contribution of treatment-related factors to infertility-specific distress. Medical procedures associated with ART, including hormonal stimulation, intensive monitoring, repeated invasive interventions, and exposure to uncertain outcomes, may themselves become significant psychological stressors [16]. Moreover, prolonged infertility duration and repeated ART cycles appear to be associated with progressively higher levels of emotional burden and psychological exhaustion [17].

### 3.2. Uncertainty and Probabilistic Thinking

Koropatnick et al. described infertility as a uniquely complex condition characterized not by the presence of pathological symptoms, but rather by the absence of a desired life event, a “non-event transition” that contributes to chronic uncertainty and difficulties in psychological adaptation [18]. Unlike many medical conditions in which diagnosis and treatment pathways may provide relatively predictable trajectories, ART is inherently characterized by uncertain outcomes, limited controllability, and repeated exposure to emotionally significant waiting periods. The success of each treatment cycle remains probabilistic rather than guaranteed, requiring patients to continuously make emotionally charged decisions under conditions of ambiguity and incomplete predictability [19]. Transactional stress theories suggest that uncertainty becomes particularly distressing when outcomes are highly relevant to personal well-being while simultaneously remaining largely uncontrollable. In ART, patients often experience a perceived lack of control over reproductive outcomes, despite intense emotional, physical, and financial investment in treatment procedures. These uncertainty-related mechanisms become particularly evident during high-uncertainty treatment phases, especially the post-transfer waiting period discussed later in the IVF trajectory [19].

### 3.3. Couple Dynamics and Dyadic Stress

Infertility and ART should not be conceptualized solely as individual stressors, but rather as shared relational experiences affecting the couple as an emotional system. Family-system and dyadic stress models suggest that partners’ psychological responses to infertility are deeply interdependent, with each partner’s emotional adjustment influencing the other’s coping processes, relational satisfaction, and overall well-being. In this perspective, infertility-related distress emerges not only from the medical condition itself, but also from the way couples collectively interpret, communicate, and manage the reproductive challenge. Several studies have shown that infertility may substantially affect multiple domains of couple functioning, including communication patterns, intimacy, sexual relationships, emotional reciprocity, and marital satisfaction. Infertility-related stress extends across different relational domains, particularly social concern, sexual concern, and relationship concern, all of which contribute to the overall emotional burden experienced during ART. Infertility stress involves both the impact of infertility on daily relational functioning and the symbolic importance attributed to parenthood and family formation. Sexual functioning may become particularly vulnerable during treatment due to scheduled intercourse, performance pressure, repeated medicalization of reproduction, and fear of disappointment following unsuccessful attempts. Couples may also progressively withdraw from social interactions because of perceived stigma, exposure to pregnancies or children within their social network, and feelings of isolation in social environments strongly centered on parenthood [20]. Importantly, psychological adjustment to infertility frequently differs between partners. Women generally report higher levels of infertility-related stress, anxiety, depressive symptoms, and emotional burden than men, whereas men may show more internalized or less openly expressed distress. These differences may partly reflect sociocultural expectations regarding gender roles and parenthood, but may also contribute to emotional asymmetry within the couple and difficulties in reciprocal understanding. Research on dyadic coping further suggests that the degree of congruence between partners’ perceptions of infertility-related stress may significantly influence psychological and relational outcomes. Peterson et al. found that couples who perceived similar levels of infertility-related stress reported higher marital adjustment, whereas incongruence between partners was associated with poorer relational functioning and greater emotional maladjustment, particularly among women. More specifically, incongruence regarding relationship concerns and the importance attributed to parenthood was associated with higher levels of female depressive symptoms. These findings support the idea that infertility-related distress should be conceptualized as a dyadic phenomenon shaped by reciprocal emotional regulation, communication quality, and shared cognitive appraisal of the infertility experience [21]. Social support also appears to play a major role in couple adjustment during ART. Perceived partner support is negatively associated with relationship concern and sexual concern, whereas supportive social networks appear to buffer infertility-related stress through more adaptive coping strategies. Conversely, inadequate emotional support, avoidant coping, and relational disengagement may contribute to worsening dyadic stress and psychological exhaustion during prolonged treatment pathways [22].

### 3.4. Male-Factor Infertility and Male Psychosocial Burden

Male-factor infertility is associated with distinct psychological and relational challenges that may not be adequately captured by models centered primarily on women undergoing ovarian stimulation and embryo transfer. Men may experience threats to masculinity, reproductive identity, sexual self-esteem, and perceived adequacy as a partner. Feelings of shame, stigma, guilt, and failure may be particularly pronounced in the presence of azoospermia, severe oligozoospermia, genetic abnormalities, erectile or ejaculatory difficulties, or the need for surgical sperm retrieval or donor sperm. The medicalization of semen production may itself generate performance pressure, embarrassment, and anxiety, particularly when sample collection occurs under time constraints or in an unfamiliar clinical environment.

Male distress may be underestimated because men frequently express psychological burden through withdrawal, irritability, work overinvolvement, problem-focused coping, sexual avoidance, or reluctance to seek support rather than through explicit reports of anxiety or sadness. Lower self-reported symptom levels should therefore not automatically be interpreted as an absence of distress. Male-factor diagnoses may also influence couple communication, sexual intimacy, decision-making concerning donor gametes, and perceptions of responsibility for treatment burden [17,18].

Male engagement should be actively facilitated rather than assuming that psychological support will be accessed spontaneously. Practical strategies include addressing both partners during consultations; offering individual and couple screening; providing male-specific written or digital information; normalizing emotional responses without feminizing the language of support; offering flexible or remote appointments; including questions about sexual functioning, shame, sample-production difficulties, and identity concerns; and involving male patients in decisions regarding treatment continuation, surgical sperm retrieval, genetic testing, and donor conception [22]. Urologists and andrologists should be included in the multidisciplinary care model alongside reproductive physicians, nurses, psychologists, psychiatrists, and genetic specialists.

### 3.5. Treatment Discontinuation and Dropout

Treatment discontinuation represents one of the most clinically relevant yet understudied outcomes in ART, as a substantial proportion of couples interrupt treatment before achieving pregnancy despite maintaining a potentially favorable prognosis. Longitudinal and epidemiological studies consistently demonstrate that dropout may occur at every stage of infertility care, from the initial diagnostic workup to repeated IVF cycles, suggesting that discontinuation should not be interpreted merely as poor adherence, but rather as the final manifestation of a complex biopsychosocial burden. A landmark systematic review by Gameiro et al., including more than 21,000 patients across 22 studies, identified psychosocial burden among the most frequent reasons for treatment discontinuation, together with relational difficulties, treatment burden, and organizational factors. Importantly, the authors emphasized that discontinuation cannot be explained solely by poor prognosis or financial limitations, but rather emerges from the reciprocal interaction between treatment-related stressors, clinic-related factors, and patient psychological vulnerability [23]. Similarly, Brandes et al., in a longitudinal cohort of subfertile couples, reported that emotional distress represented the leading reason for withdrawal from fertility care, followed by poor prognosis and rejection of further treatment. Notably, approximately half of discontinuations occurred before initiation of fertility treatment and nearly one-third after at least one IVF cycle, highlighting how treatment burden may progressively accumulate throughout the ART pathway [24]. The central role of psychological distress is further supported by studies conducted in healthcare systems with substantial financial coverage, where dropout rates remain unexpectedly high despite reduced economic barriers. Rajkhowa et al. demonstrated that psychological stress and repeated treatment failure were among the strongest determinants of discontinuation, with psychological stress being increasingly reported after multiple unsuccessful attempts [25]. Likewise, Domar et al. found that stress was the primary reason insured patients in the United States prematurely terminated IVF treatment, with relationship strain and anxiety/depressive symptoms representing the most frequently reported contributors [26]. These dimensions of infertility-related distress are not static, but dynamically evolve across different phases of the ART pathway.

## 4. The IVF Pathway as a Psychological Trajectory

**Figure 1:**
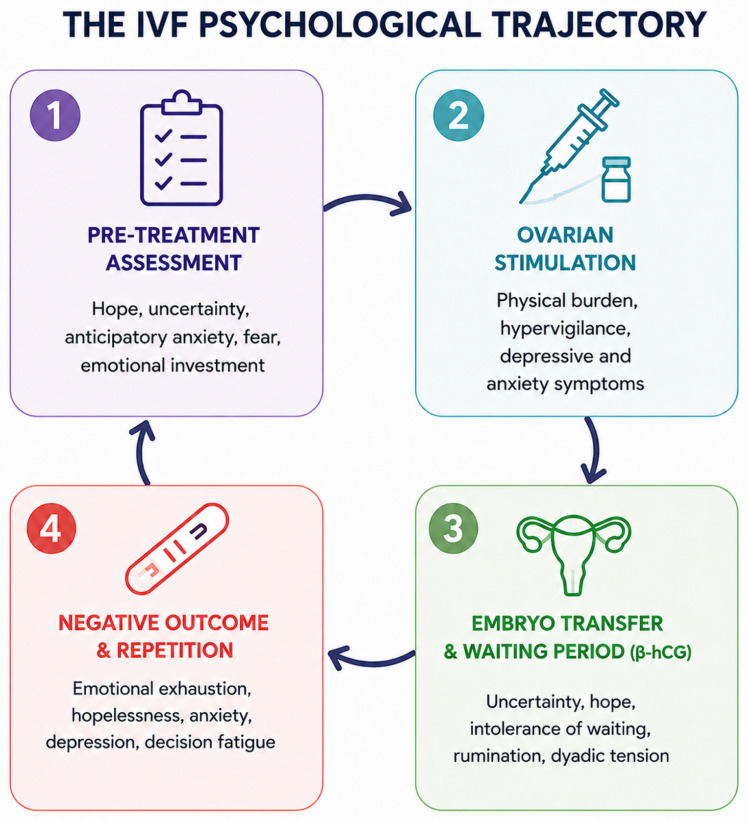
Cyclical representation of the IVF emotional responses across treatment phases. Note. This schematic illustration depicts the nonlinear psychological journey of patients undergoing assisted reproductive technology. The trajectory is divided into four critical thematic stages: the initial pretreatment assessment dominated by anticipatory anxiety and emotional investment, the ovarian stimulation phase characterized by physical burden and hypervigilance, the embryo transfer and waiting period involving significant intolerance of uncertainty and dyadic tension, and the negative outcome phase leading to emotional exhaustion and decision fatigue. The model highlights how psychological distress is not a static condition but a fluctuating experience that accumulates through repeated cycles, requiring a dynamic and phase-specific approach to psychosocial care to address the evolving emotional needs of the patient and the couple.

The IVF pathway is not merely a medical procedure but a trajectory that exposes patients to multiple salient psychological phases, each associated with specific stressors [27]. Different components of treatment evoke distinct emotional reactions, including anxiety during active procedures and depressive symptoms following unsuccessful outcomes. Rather than remaining stable over time, psychological distress fluctuates across treatment stages and may progressively accumulate following repeated unsuccessful cycles [28]. Accordingly, emotional adjustment during IVF is strongly influenced by both the treatment phase and treatment outcome (Figure 1).

### 4.1. Pre-Treatment Phase

The pre-treatment phase is frequently characterized by anticipatory anxiety, heightened emotional investment, and uncertainty regarding both prognosis and treatment success. At this stage, many patients enter fertility care after prolonged periods of unsuccessful attempts to conceive, previous miscarriages, recurrent pregnancy losses, or prior failed fertility treatments, experiences that may already have generated significant emotional burden before ART initiation [29]. Patients may simultaneously experience fear related to treatment failure, invasive procedures, financial burden, and the potential definitive loss of biological parenthood. The beginning of IVF treatment may additionally intensify the medicalization of reproduction. Couples frequently transition from perceiving conception as a private and spontaneous process to experiencing it as a highly monitored and technologically mediated medical pathway involving strict schedules, hormonal protocols, and repeated clinical evaluations [30]. This transition may alter perceptions of bodily autonomy, intimacy, and reproductive identity even before treatment procedures begin. At the same time, patients often develop extremely high emotional expectations regarding IVF success, particularly when ART is perceived as the “last available option” after prolonged infertility. Unrealistic expectations and excessive emotional investment may increase vulnerability to subsequent emotional deterioration following unsuccessful cycles. These findings support the importance of early psychosocial screening prior to treatment initiation.

### 4.2. Ovarian Stimulation

ART protocols typically require controlled ovarian hyperstimulation, serial ultrasonographic assessments, blood tests, strict medication schedules, and oocyte retrieval procedures, all of which may contribute to heightened bodily awareness and somatic hypervigilance [31,32]. During this phase, patients often become increasingly focused on physical sensations and treatment indicators, interpreting abdominal discomfort, pelvic tension, breast tenderness, or other bodily changes as potential predictors of treatment success or failure. This hypervigilant monitoring of bodily states may further amplify emotional activation and perceived loss of control. Recent evidence additionally suggests that emotional distress during ovarian stimulation may not be exclusively attributable to the psychological burden of infertility treatment itself, but may also partially reflect direct neuroendocrine effects of rapid hormonal fluctuations induced by ART protocols. Protocols involving gonadotropin stimulation and GnRH agonists appear to be associated with less favorable mood profiles compared with natural-cycle or antagonist-based protocols, with several studies showing mild but consistent worsening of depressive symptoms during treatment. Ovarian suppression and hypoestrogenic states induced by GnRH agonists may contribute to increased vulnerability to depressive symptoms through dysregulation of hypothalamic-pituitary-gonadal axis functioning [33]. Depressive and anxiety symptoms may progressively increase from baseline through the estradiol-dominant stimulation phase and remain elevated during the progesterone-dominant phase following embryo transfer. Importantly, worsening depressive symptoms appear to be associated not with absolute estradiol concentrations, but rather with abrupt declines in estradiol levels occurring between stimulation and post-transfer phases, suggesting that rapid hormonal fluctuations themselves may act as affective destabilizers in vulnerable women. Individual sensitivity to reproductive hormonal changes, rather than hormonal imbalance alone, may therefore underlie susceptibility to mood symptoms during ART treatment [34].

### 4.3. Embryo Transfer and the Waiting Period

The period between embryo transfer and pregnancy testing represents one of the most psychologically demanding phases of the IVF trajectory. During this interval, patients are exposed to intense uncertainty while simultaneously having minimal ability to influence treatment outcomes. Medical waiting periods may be experienced as uniquely stressful because outcomes are highly consequential, unpredictable, and largely uncontrollable. In IVF, the “two-week wait” is often experienced as a suspended psychological state in which hope and fear continuously alternate while awaiting pregnancy test results. The inability to obtain objective information regarding implantation success may further intensify uncertainty and preoccupation. These feelings promote rumination, intrusive thoughts, emotional ambivalence, and hypervigilance toward bodily sensations potentially interpreted as signs of implantation or treatment failure. Patients often repeatedly reinterpret normal physiological sensations as meaningful reproductive indicators, engaging in constant symptom monitoring, reassurance-seeking behaviors, and excessive internet searching for predictive signs of pregnancy success or failure. Persistent rumination regarding the possibility of pregnancy has been associated with levels of intrusive ideation comparable to those observed in patients attending stress clinics. Waiting periods become particularly stressful when individuals perceive outcomes as highly meaningful but largely uncontrollable. In these circumstances, problem-focused coping strategies become less effective, while patients increasingly rely on emotion-focused or meaning-based coping mechanisms aimed at regulating distress rather than altering the situation itself [19,20,21,22,23,24,25,26,27,28,29,30,31,32,33,34,35]. Although brief coping interventions may not necessarily reduce anxiety levels, they appear capable of increasing positive affect and perceived psychological coping during the waiting period [36]. Intolerance of uncertainty represents a central psychological mechanism linking infertility-related stress to more severe psychopathological outcomes. In women undergoing frozen-thawed embryo transfer after previous early pregnancy loss, elevated intolerance of uncertainty has been strongly associated with post-traumatic stress symptoms, hyperarousal, emotional dysregulation, and cognitive distress [37]. The repeated exposure to uncertain reproductive outcomes may therefore contribute not only to transient anxiety, but also to persistent trauma-related psychological distress in a subgroup of patients. This phase may also affect interpersonal functioning. Couples may adopt different coping strategies while waiting for treatment outcomes, with some preferring emotional sharing and reassurance, whereas others rely on emotional distancing or avoidance in an attempt to protect themselves from possible disappointment. These differences may occasionally contribute to dyadic tension and communication difficulties.

### 4.4. Cycle Failure and Repetition

Unsuccessful treatment cycles frequently represent critical psychological turning points within the ART trajectory. While many patients initially maintain high levels of hope and motivation, repeated failures may progressively contribute to emotional exhaustion, hopelessness, grief reactions, demoralization, and loss of perceived reproductive control. Negative emotional responses may significantly increase following unsuccessful IVF cycles and persist across repeated treatment attempts [27,28,29,30,31,32,33,34,35,36,37,38]. Cycle failure may reactivate the multidimensional grief processes associated with infertility, including mourning for anticipated parenthood, disruption of future life plans, diminished self-esteem, and fear of permanent childlessness [29]. Following repeated unsuccessful cycles, some patients progressively shift from acute emotional reactions toward more chronic negative emotional state. Recent evidence further suggests that infertility treatment failure may generate prolonged grief responses comparable, in some patients, to those observed after other major reproductive losses. Women undergoing unsuccessful ART frequently report despair, loss of hope, guilt, anger, frustration, anxiety, and social isolation, while some studies also describe suicidal ideation and profound existential suffering. The emotional impact of ART failure may also remain persistent over time rather than resolving immediately after treatment discontinuation, with subsequent reduced treatment adherence. Although distress may partially decrease over time, anxiety and depressive symptoms often remain elevated above baseline levels even six months after failed treatment. This process may be seen as a progressive “self-reconstruction” in which women repeatedly renegotiate their identity, emotional investment, and future life perspectives following unsuccessful ART attempts [38,39]. ART failure may also contribute to maladaptive coping behaviors, including treatment avoidance, social withdrawal, denial, and emotional disengagement. In severe cases, persistent emotional dysregulation may compromise interpersonal functioning, occupational performance, and overall quality of life. Importantly, repeated ART cycles may also generate cumulative decision fatigue, as couples are repeatedly required to make emotionally and financially demanding decisions under uncertain prognostic conditions. Decisions regarding continuation of treatment, use of additional IVF cycles, gamete donation, embryo preservation, or treatment discontinuation may progressively become more psychologically burdensome over time and contribute to treatment exhaustion and disengagement from fertility care pathways [40].

## 5. Screening and Risk Stratification

The high prevalence and heterogeneous presentation of psychological distress among individuals undergoing ART highlight the need for systematic psychosocial screening within fertility care pathways. Psychological burden in infertility is not uniformly distributed across patients and may range from transient emotional distress to severe psychiatric symptomatology associated with significant functional impairment, relational difficulties, and treatment discontinuation. Consequently, psychosocial assessment in ART should not be limited to the identification of overt psychiatric disorders, but should also include the evaluation of infertility-specific quality of life, relational functioning, coping resources, and vulnerability factors that may influence both psychological adjustment and treatment adherence [38,39,40,41]. Recent evidence increasingly supports the integration of routine mental health screening into standard fertility care pathways. A growing body of literature suggests that psychosocial assessment should not be considered ancillary to reproductive treatment, but rather an essential component of comprehensive infertility care [12]. The European Society of Human Reproduction and Embryology (ESHRE) specifically recommends routine psychosocial care throughout infertility treatment, including the assessment of emotional, relational, behavioral, and cognitive needs before, during, and after ART procedures [42]. Longitudinal and phase-specific psychosocial screening models have therefore been proposed across different treatment phases rather than isolated assessments performed only at treatment initiation. Repeated evaluation may allow clinicians to identify dynamic changes in emotional burden occurring across ovarian stimulation, embryo transfer, waiting periods, and repeated treatment failures [16]. In this context, risk stratification models may help fertility clinics move from generic supportive approaches toward more individualized psychosocial care pathways.

### 5.1. Validated Screening Instruments

A growing number of validated psychometric instruments are currently available for the assessment of psychological distress and quality of life in infertile individuals undergoing ART. These tools can be broadly divided into infertility-specific instruments and general psychiatric screening measures. Among infertility-specific instruments, the Fertility Quality of Life questionnaire (FertiQoL) is currently considered the most widely used and best validated tool for assessing infertility-related quality of life. Developed through international collaboration between the ESHRE and the American Society for Reproductive Medicine (ASRM), the FertiQoL was specifically designed to evaluate the impact of infertility and fertility treatments on emotional, relational, social, and mind–body functioning in both women and men experiencing fertility problems. The instrument includes a Core module assessing emotional, physical, relational, and social domains, as well as an optional Treatment module exploring treatment tolerability and environmental aspects of fertility care [43]. Subsequent psychometric studies confirmed satisfactory reliability and validity of the FertiQoL across different cultural settings and patient populations. A recent systematic review analyzing the psychometric properties of the instrument concluded that FertiQoL demonstrates adequate internal consistency, structural validity, and convergent validity with measures of anxiety, depression, and general quality of life, supporting its use as a reliable infertility-specific outcome measure in both clinical and research settings [44]. The questionnaire also appears particularly useful for identifying domains of psychosocial impairment that may not be adequately captured by generic psychiatric scales, including relational strain, social isolation, sexual difficulties, and treatment-related burden [45]. Specific attention has also been directed toward the relational dimension of infertility-related quality of life. The FertiQoL Relational subscale demonstrated good psychometric properties and significant correlations with dyadic adjustment, marital satisfaction, and infertility-related distress, supporting its utility in identifying couples at higher risk of relational deterioration during ART treatment [46]. Alongside infertility-specific measures, brief transdiagnostic screening instruments such as the Hospital Anxiety and Depression Scale (HADS), Patient Health Questionnaire-9 (PHQ-9), and Generalized Anxiety Disorder-7 (GAD-7) are frequently used in fertility settings to rapidly identify clinically relevant depressive and anxiety symptoms. These instruments are particularly useful because of their brevity, ease of administration, and established diagnostic thresholds. Although they do not specifically assess infertility-related distress, they may facilitate the early identification of patients requiring more comprehensive psychiatric or psychological evaluation. In particular, PHQ-9 and GAD-7 may help identify patients with moderate-to-severe depressive or anxiety symptoms, suicidal ideation, or persistent emotional dysregulation requiring referral to specialized mental health care. Beyond infertility-specific quality-of-life assessment, several studies have emphasized the clinical utility of structured psychosocial screening instruments specifically designed to identify patients at risk for emotional maladjustment during ART. Among these, SCREENIVF represents one of the most extensively studied screening tools in reproductive medicine. SCREENIVF is a 34-item multidimensional questionnaire developed to identify women at increased risk for psychological maladjustment before IVF/ICSI treatment through the evaluation of five major vulnerability domains: pretreatment anxiety, pretreatment depression, helplessness cognitions, low acceptance of fertility problems, and poor perceived social support. Validation studies demonstrated acceptable psychometric performance of SCREENIVF, with sensitivity and specificity values of 69% and 77%, respectively, and a particularly high negative predictive value for identifying patients not at risk of subsequent emotional maladjustment after unsuccessful ART cycles. Importantly, the instrument was specifically developed to facilitate early identification of emotionally vulnerable patients before treatment initiation, thereby supporting timely referral to psychosocial support when needed [47]. Additional evidence also supports the feasibility of implementing systematic psychosocial screening in routine fertility practice. In a prospective cohort study evaluating the integration of SCREENIVF into daily IVF clinical care, uptake rates remained stable at approximately 78–80%, while most patients considered the screening process useful and acceptable. Approximately one-third of screened patients were identified as being at risk for emotional maladjustment before treatment initiation. However, several barriers to help-seeking behavior were also identified, including limited perceived need for psychological support, stigma, and traveling distance to mental health services [48]. More recent cross-cultural validation studies further confirmed the reliability and clinical applicability of SCREENIVF in different healthcare settings. The Hungarian validation study demonstrated good construct validity, high internal consistency, and significant correlations between SCREENIVF scores and infertility-related quality of life assessed through FertiQoL [42]. These findings support the cross-cultural robustness of infertility-specific psychosocial screening approaches. Additional instruments, including the Fertility Problem Inventory (FPI), Fertility Problem Stress questionnaire (FPS), and Illness Cognitions Questionnaire adapted for infertility (ICQ-I), may provide further information regarding infertility-specific stress, maladaptive cognitions, and coping styles. However, compared with FertiQoL, these tools currently show more limited evidence regarding psychometric robustness, cross-cultural validation, or clinical applicability [45]. Although validated instruments may facilitate the identification of psychological vulnerability during ART, their clinical usefulness depends on the definition of appropriate administration time points, interpretative thresholds, referral criteria, and reassessment intervals. Screening scores should not be considered diagnostic in isolation or used as automated determinants of treatment eligibility. Rather, they should function as clinical triggers for further assessment and should be interpreted together with psychiatric history, functional impairment, reproductive experiences, couple functioning, treatment phase, and patient preferences. Table 1 summarizes a proposed operational approach to the use of the principal psychosocial screening instruments in ART settings.

As shown in Table 1, no single instrument captures the full spectrum of psychological needs associated with infertility and ART. General symptom measures, such as the PHQ-9, GAD-7, and HADS, may identify clinically relevant anxiety or depressive symptoms, whereas SCREENIVF and FertiQoL provide more specific information concerning emotional maladjustment and infertility-related quality of life. The choice of instrument should therefore reflect the clinical purpose of the assessment. Positive screening results should prompt direct clinical evaluation rather than automatic assignment to a risk category, particularly when suicidal ideation, severe functional impairment, trauma-related symptoms, or marked relational deterioration are identified.

### 5.2. Risk Profiles

Psychological vulnerability during ART is heterogeneous, and the categories proposed below are intended as pragmatic clinical groupings rather than validated prognostic classes. They have not yet been assigned empirically derived cut-offs, nor have their calibration, discrimination, or predictive validity been prospectively established. Classification should therefore combine validated screening scores with clinical interview, functional impairment, psychiatric history, reproductive history, couple functioning, patient preference, and current treatment phase. The framework is intended to guide proportional care and reassessment rather than determine access to reproductive treatment through an automated score.

Risk stratification models may help identify patients with different levels of psychological vulnerability and facilitate the implementation of targeted interventions proportional to clinical needs. Several studies suggest that vulnerability to emotional maladjustment during ART depends on the interaction between pre-existing psychological characteristics, infertility-specific cognitions, treatment-related stressors, and relational or sociocultural factors. Women characterized by elevated baseline anxiety or depressive symptoms, low perceived social support, strong helplessness cognitions, or reduced acceptance of infertility appear particularly vulnerable to persistent relevant emotional maladjustment following unsuccessful treatment [47,48]. A low-risk profile may include patients presenting transient emotional distress directly associated with infertility diagnosis or specific treatment phases, while overall psychological functioning, relational stability, and coping resources remain relatively preserved. These individuals generally show adaptive coping strategies, adequate social support, preserved quality of life, and no history of major psychiatric disorders. Emotional symptoms in this group are often situational and tend to fluctuate according to treatment phases without significant long-term functional impairment. Moderate-risk profiles may instead include patients experiencing persistent anxiety, elevated infertility-related distress, relational strain, reduced self-efficacy, maladaptive cognitive styles, or increasing emotional exhaustion across repeated treatment cycles. Previous treatment failures, prolonged infertility duration, intolerance of uncertainty, financial burden, and occupational conflicts may further contribute to emotional deterioration in this subgroup [16]. Studies investigating infertility-related quality of life additionally suggest that socioeconomic factors, educational level, perceived infertility cause, and infertility duration may significantly influence emotional adjustment and psychosocial functioning during ART [49,50]. High-risk profiles may include individuals presenting current or previous psychiatric disorders, trauma histories, recurrent pregnancy losses, severe depressive or anxiety symptoms, suicidal ideation, significant relational dysfunction, or marked emotional dysregulation during treatment. Repeated ART failure appears particularly associated with persistent elevations in anxiety and depressive symptoms, reduced marital satisfaction, and impaired overall well-being [38]. Additional risk factors identified in recent studies include poor family emotional support, severe financial burden, social stigma, prolonged treatment duration, and repeated exposure to unsuccessful ART cycles [16]. Gender differences may also influence psychological risk profiles. Several studies consistently report lower fertility-related quality of life and higher depressive symptom scores among women compared with men, particularly regarding emotional and mind–body domains [50,51]. However, male psychological distress may be underrecognized due to differences in emotional expression, coping styles, and help-seeking behaviors.

### 5.3. Proposed Conceptual Stratification Framework

Given the multidimensional and fluctuating nature of distress during ART, a stepped and longitudinal stratification framework may provide a clinically useful structure for organizing psychosocial care. The framework is conceptual and should not be regarded as a validated risk-prediction algorithm.

An initial psychosocial screening could be performed before ART initiation using a combination of infertility-specific and general psychiatric instruments, such as FertiQoL together with PHQ-9, GAD-7, or HADS. This first assessment should aim to identify baseline psychiatric vulnerability, infertility-related distress, relational functioning, coping resources, and previous traumatic reproductive experiences. Based on this initial assessment, patients may be stratified according to their level of psychological vulnerability and directed toward appropriate psychosocial interventions during ART treatment (Table 2).

Patients classified as low risk may primarily benefit from universal supportive interventions, including psychoeducation, emotional normalization, stress-management information, and access to supportive counseling when needed. Periodic reassessment during major treatment phases may nonetheless remain important to detect worsening distress following unsuccessful cycles or prolonged treatment duration. Patients presenting moderate-risk profiles may require more structured psychological support, including cognitive-behavioral interventions, stress-management approaches, infertility-focused counseling, or couple-based interventions targeting communication difficulties and dyadic coping. Reassessment after failed cycles or during emotionally demanding phases such as embryo transfer waiting periods may be particularly important in this subgroup. High-risk patients may instead require integrated multidisciplinary management involving reproductive physicians, fertility nurses, psychologists, psychiatrists, urologists or andrologists, genetic specialists, and other professionals according to individual clinical needs. In these cases, fertility treatment pathways may need to incorporate formal psychiatric evaluation, trauma-informed interventions, pharmacological treatment when indicated, and close longitudinal monitoring of emotional safety and treatment adherence. Emerging expert perspectives increasingly advocate for mental health screening and psychosocial support to become standard components of fertility care rather than optional adjunctive services. Despite the high prevalence of clinically significant anxiety and depressive symptoms among infertility patients, utilization of mental health services remains low due to barriers including stigma, financial burden, limited accessibility, travel distance, and insufficient awareness regarding available psychological support [12,48]. Consequently, integrated and proactive psychosocial screening models may represent an important strategy to improve early identification of vulnerable patients and facilitate timely referral to supportive interventions.

### 5.4. Safety Pathway for Suicidal Ideation and Acute Psychiatric Risk

Any disclosure of suicidal ideation, self-harm intent, recent suicidal behavior, severe hopelessness, psychotic symptoms, mania, or inability to maintain personal safety requires immediate clinical escalation and should not be managed solely through a screening questionnaire or digital platform. A positive response to a self-harm item should be followed by a direct, same-day assessment of current thoughts, intent, plan, access to means, previous attempts, substance use, protective factors, available support, and capacity to participate in safety planning.

When imminent or high suicide risk is identified, the patient should not be left alone, and emergency psychiatric assessment should be arranged according to local procedures. Documentation should include the screening result, clinical assessment, actions taken, professionals contacted, communication with the patient and, with consent or when legally required, supportive others, and the agreed follow-up plan. The fertility and mental-health teams should establish a named clinician responsible for coordination and ensure active follow-up after emergency assessment or discharge.

The presence of psychological distress or a psychiatric diagnosis should not automatically exclude a patient from ART [14,16]. Decisions regarding continuation or temporary deferral of a cycle should be individualized and made jointly by reproductive and mental-health professionals. Temporary deferral may be appropriate when acute psychiatric instability compromises safety, decisional capacity, adherence to essential treatment instructions, or the ability to provide informed consent. Treatment should be reconsidered promptly after stabilization, with explicit criteria for re-entry and efforts to minimize stigma or punitive interpretations.

## 6. Targeted Interventions Across the IVF Pathway

Contemporary evidence indicates that psychological interventions can improve anxiety, depressive symptoms, infertility-related distress, coping, and quality of life. Evidence concerning reproductive endpoints is considerably less certain because studies vary in design, intervention content, participant prognosis, and control of reproductive confounders. Accordingly, psychosocial interventions should be recommended primarily for their patient-centered psychological and relational benefits, while possible effects on adherence, dropout, pregnancy, or live birth should be regarded as secondary and not definitively established [10,52].

Recent systematic reviews consistently demonstrate that psychosocial interventions exert beneficial effects on anxiety, depressive symptoms, infertility-related stress, and emotional adjustment during fertility treatment [10,53,54]. Nonetheless, not all patients appear to benefit equally, and growing evidence supports the implementation of stepped and stratified intervention models tailored to individual psychological vulnerability profiles, infertility-specific stressors, relational functioning, and treatment phase (Table 3).

### 6.1. Universal Interventions

Universal psychosocial interventions represent low-intensity supportive strategies that may be offered routinely to all patients undergoing fertility treatment, regardless of baseline psychological risk. These interventions primarily aim to normalize emotional reactions, improve understanding of infertility-related stress responses, reduce stigma, and enhance patients’ perceived sense of preparedness and emotional control across different stages of fertility treatment. Psychoeducation constitutes one of the most widely recommended universal interventions in reproductive medicine. Educational approaches generally focus on providing information regarding infertility-related emotional reactions, treatment procedures, realistic success expectations, stress responses, coping strategies, and available psychosocial resources. Early literature suggested that interventions emphasizing education and skills training may be particularly effective in reducing negative affect among infertile patients [55]. Educational interventions may additionally reduce uncertainty and anticipatory anxiety by improving patients’ understanding of treatment phases and normalizing emotional fluctuations commonly experienced during ART. Stress normalization also represents an important component of universal psychosocial care. Infertility and ART frequently expose patients to repeated uncertainty, prolonged waiting periods, bodily monitoring, and emotionally salient outcomes. Consequently, many patients interpret their emotional reactions as signs of personal inadequacy or psychological dysfunction. Normalization-based interventions may help patients recognize anxiety, sadness, ambivalence, grief, or emotional exhaustion as understandable responses to a demanding reproductive experience rather than indicators of psychopathology. Current expert perspectives increasingly advocate for the integration of routine psychosocial support into standard fertility care pathways rather than limiting psychological care to severe psychiatric presentations [12]. Universal psychosocial support may additionally facilitate earlier identification of vulnerable individuals who require more structured interventions during later treatment phases.

### 6.2. Structured Psychological Therapies

Patients presenting persistent emotional distress, maladaptive cognitions, impaired coping flexibility, or clinically significant anxiety and depressive symptoms may benefit from more structured psychotherapeutic interventions. Among the available approaches, cognitive behavioral therapy (CBT) currently represents the most extensively studied psychological intervention in infertility care. CBT interventions for infertility generally target maladaptive cognitive patterns associated with infertility-related suffering, including catastrophic interpretations, helplessness cognitions, excessive self-blame, perfectionistic expectations, hypervigilance toward bodily symptoms, and persistent treatment-related rumination. Meta-analytic evidence suggests that CBT significantly reduces depression, anxiety, psychological distress, perceived stress, and infertility-specific stress while improving quality of life among women undergoing infertility treatment [53]. Additional meta-analyses support the beneficial effects of psychotherapy, particularly CBT-based approaches, on psychological outcomes. Some studies have also reported higher pregnancy rates among intervention participants; however, these findings are heterogeneous and potentially influenced by age, infertility diagnosis, previous treatment, treatment exposure, study attrition, and baseline reproductive prognosis. Current evidence is therefore insufficient to conclude that CBT independently improves pregnancy or live-birth rates [10,52]. Several mechanisms may explain the efficacy of CBT during ART treatment. Cognitive restructuring may reduce maladaptive appraisals regarding treatment failure and reproductive uncertainty, while behavioral interventions may improve stress regulation, emotional self-efficacy, and coping flexibility. Some reviews additionally suggest that CBT may exert beneficial neurophysiological effects by modulating stress-related activation of hypothalamic–pituitary–adrenal and hypothalamic-pituitary-gonadal pathways involved in reproductive functioning. Proposed biological mechanisms include modulation of stress-related neuroendocrine activity, but these pathways remain hypothetical and have not established a causal link between psychotherapy and improved reproductive outcomes [56]. Alongside CBT, increasing attention has been directed toward acceptance-based and mindfulness-informed interventions. Acceptance and Commitment Therapy (ACT) appears particularly relevant in infertility settings because ART often involves prolonged exposure to uncertainty, unpredictability, and partially uncontrollable outcomes. Rather than attempting to eliminate distress entirely, ACT-based approaches emphasize psychological flexibility, acceptance of difficult emotional experiences, cognitive defusion, and value-based adaptation despite ongoing uncertainty. Recent reviews indicate that ACT-based interventions may improve emotional adjustment, coping abilities, and quality of life among infertile individuals [57]. Self-compassion and mindfulness-oriented approaches have also shown promising preliminary results. In randomized and pilot studies, self-compassion-based interventions delivered through videos, digital storytelling, or self-guided mindfulness programs significantly reduced anxiety and depressive symptoms while improving infertility self-efficacy, self-compassion, and fertility-related quality of life [58,59]. Although current evidence generally supports the efficacy of structured psychotherapies in infertility care, effect sizes remain variable across studies and geographical regions, while treatment accessibility and long-term adherence continue to represent major clinical challenges [10]. These findings reinforce the need for more individualized and scalable intervention models.

### 6.3. Couple-Based Interventions

Given the inherently relational nature of infertility and ART, increasing attention has been directed toward couple-based psychological interventions. Recent actor–partner interdependence models further demonstrated that fertility stress, dyadic coping, and marital quality show significant reciprocal associations within IVF couples, with one partner’s distress potentially influencing the other partner’s relational adjustment and psychological functioning [60]. Consequently, interventions exclusively targeting individual psychopathology may inadequately address important relational dimensions of infertility-related distress. Emerging couple-based frameworks additionally suggest that coping strategies adopted by one partner may directly influence the psychological adjustment of the other partner, highlighting the limitations of exclusively individual-centered psychosocial interventions in infertility care [61]. Couple-based interventions generally aim to improve communication, emotional reciprocity, mutual support, conflict management, and dyadic coping abilities. Systematic reviews suggest that couple therapy may effectively reduce anxiety, depression, stress, and relational dissatisfaction among couples undergoing infertility treatment, particularly when interventions include six or more sessions and integrate systemic, attachment-based, or cognitive-behavioral components [62]. Dyadic coping represents one of the most clinically relevant relational constructs in ART settings. Positive dyadic coping behaviors, including emotional validation, collaborative problem-solving, delegated coping, and supportive communication, appear to be associated with better marital adjustment and reduced infertility-related distress [63]. Dyadic coping has also been shown to mediate the relationship between fertility stress and marital quality during IVF treatment, supporting the hypothesis that couple functioning may represent both a vulnerability factor and a protective resource during ART [60]. Conversely, negative dyadic coping patterns characterized by withdrawal, hostility, avoidance, or ineffective stress communication may contribute to relational deterioration during treatment. Couple functioning may additionally influence treatment adjustment and reproductive outcomes. Higher levels of relational cohesion, flexibility, and communication quality have been associated with lower psychological symptomatology during ART treatment, whereas relational dysfunction may contribute to emotional deterioration and reduced treatment adherence [64]. These findings further support the integration of systemic and couple-oriented perspectives into psychosocial infertility care. Male engagement may be improved through low-stigma, practical, and flexible models of care. Fertility teams should address male partners directly during consultations rather than positioning them solely as supporters of the female patient. Screening and intervention materials should include male-factor concerns such as shame, sexual functioning, sample-production anxiety, azoospermia, surgical sperm retrieval, genetic risk, and donor sperm. Brief individual consultations, evening or telehealth appointments, digital self-assessment, couple-based sessions, and collaboration with urologists or andrologists may increase acceptability. Clinicians should also recognize that withdrawal, irritability, overinvestment in work, or excessive problem-solving may represent manifestations of distress even when men do not report sadness or anxiety explicitly.

### 6.4. Digital and Scalable Solutions

Despite the growing evidence supporting psychosocial interventions in infertility care, access to specialized mental health services remains limited for many patients because of cost, geographical barriers, stigma, limited availability of trained professionals, and treatment-related time constraints. Consequently, increasing attention has been directed toward digital and scalable psychological interventions capable of improving accessibility and continuity of care. Recent studies suggest that internet-based and e-health interventions may effectively reduce infertility-related distress, anxiety, and depressive symptoms while improving quality of life and coping abilities [65,66]. Telemedicine-based psychological programs have employed multiple technological formats, including web-based CBT modules, ACT interventions, positive reappraisal training, mobile applications, digital storytelling, self-administered manuals, online videos, and smartphone-delivered coping exercises [67]. Internet-based CBT interventions specifically adapted for infertility have shown encouraging preliminary results. Fully automated programs integrating individualized text, audio, and video components significantly improved infertility-related stress, anxiety, depressive symptoms, and fertility-related quality of life while demonstrating high levels of patient acceptability and satisfaction [68,69]. Notably, larger treatment effects appear to occur among individuals presenting poorer baseline quality of life or clinically significant emotional symptoms, supporting the potential utility of risk-stratified digital interventions. Mobile and smartphone-supported interventions additionally offer the possibility of delivering phase-specific coping strategies during highly stressful treatment phases, such as ovarian stimulation or the embryo transfer waiting period. Smartphone-based positive adjustment coping interventions and self-compassion programs have demonstrated good feasibility and acceptability, although effects on anxiety and depressive symptoms remain heterogeneous across studies [58,59,60,61,62,63,64,65,66,67,68,69,70]. Digital mental health interventions should not be considered merely as substitutes for traditional psychotherapy, but rather as potentially complementary tools within stepped-care and precision psychosocial models. Qualitative evidence further suggests that patients particularly value psychological interventions that provide individualized emotional support, practical coping strategies, and clearer educational guidance regarding ART procedures and uncertainty management. Participants also emphasized the importance of couple-oriented interventions and continuity of psychological support following unsuccessful treatment outcomes [71]. Low-intensity digital interventions may provide scalable support for low- and moderate-risk patients, facilitate early symptom monitoring, and improve access to care, while more complex psychiatric or relational presentations may still require integrated multidisciplinary management involving specialized mental health professionals.

### 6.5. Ethical, Legal, and Data-Governance Considerations for Digital Psychosocial Care

Digital screening, telepsychology, and app-based monitoring may improve accessibility, but their implementation requires clear governance. Patients should receive transparent information regarding what data are collected, where they are stored, who can access them, how long they are retained, and whether they are used for research, profiling, or algorithm development. Consent for digital monitoring should be specific and should not be presented as a condition for receiving fertility treatment.

Automated scores or algorithmic alerts should support rather than replace clinical judgment. Digital systems may perform differently across sex, gender, age, language, socioeconomic, and cultural groups; therefore, potential algorithmic bias and unequal access must be evaluated. Clinics should define who is responsible for reviewing alerts, the maximum response time, procedures for out-of-hours notifications, and the action required when suicidal ideation or other high-risk content is detected. Applications without an active crisis-response pathway should not imply real-time monitoring.

Confidentiality is especially important because reproductive and mental-health data are highly sensitive. Platforms should comply with applicable data-protection requirements, use proportionate security safeguards, minimize data collection, and maintain audit trails. Clinical liability, escalation responsibilities, and communication between technology providers, fertility professionals, and mental-health clinicians should be defined before implementation [1,3]. Digital care should remain optional and should not replace face-to-face assessment when clinical complexity, safety concerns, digital exclusion, or patient preference makes direct care more appropriate.

## 7. Clinical Integration and Interdisciplinary Collaboration

**Figure 2:**
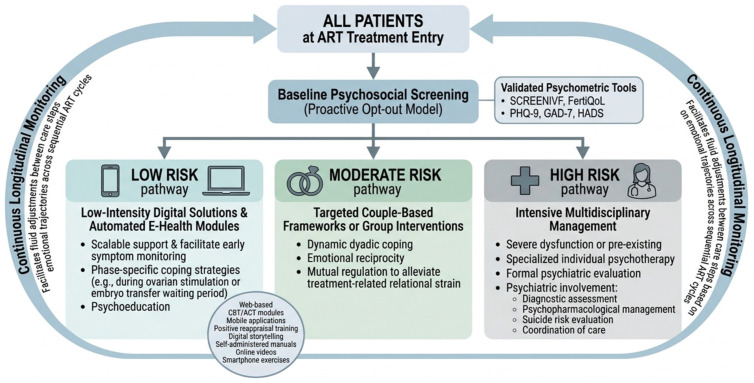
Stratified stepped care screening and psychosocial intervention algorithm. Note. This clinical triage flowchart illustrates the proactive opt-out assessment model derived from integrated fertility care frameworks. All patients undergo baseline psychosocial screening utilizing validated psychometric tools at treatment entry to determine their specific risk profile. Low-risk presentations transition to low-intensity digital solutions and automated e-health modules that provide scalable support, phase-specific coping strategies, and psychoeducation. Moderate risk profiles receive targeted couple-based frameworks or group interventions focused on dynamic dyadic coping, emotional reciprocity, and mutual regulation to alleviate treatment-related relational strain. High-risk patients presenting severe dysfunction or pre-existing trauma are directly routed to intensive multidisciplinary management involving specialized individual psychotherapy and formal psychiatric evaluation. The architecture incorporates continuous longitudinal monitoring to facilitate fluid adjustments between care steps based on emotional trajectories across sequential assisted reproductive technology cycles.

The ESHRE explicitly defines psychosocial care as care aimed at helping patients optimize fertility treatment while managing the psychological and social implications of infertility and its treatment. Current biopsychosocial and patient-centered models emphasize that infertility affects biological, psychological, relational, social, and behavioral dimensions simultaneously, requiring coordinated collaboration between reproductive specialists, psychologists, psychiatrists, nurses, counselors, embryologists, and administrative staff. Integrated fertility care therefore aims not only to maximize reproductive outcomes but also to reduce treatment burden, improve emotional well-being, facilitate treatment adherence, and support patients throughout the entire ART pathway [72,73]. Psychiatrists may play a particularly important role in the management of patients presenting severe or persistent psychological symptoms during infertility treatment. While many patients experience subclinical emotional distress, a clinically significant subgroup develops anxiety disorders, depressive disorders, trauma-related symptoms, suicidal ideation, or severe emotional dysregulation requiring specialized psychiatric evaluation and treatment. Psychiatric involvement may be especially relevant in patients with pre-existing psychiatric disorders, previous trauma histories, recurrent pregnancy loss, severe infertility-related grief, marked functional impairment, or poor treatment adherence. In these cases, psychiatrists may contribute to diagnostic assessment, psychopharmacological management, suicide risk evaluation, and coordination of care with reproductive specialists and psychologists. Particular attention may also be required regarding the safety and risk-benefit balance of psychotropic medications during ovarian stimulation, pregnancy attempts, and pregnancy itself. Recent expert perspectives increasingly advocate for mental health screening to become a standard component of fertility care, ideally beginning at treatment intake and continuing longitudinally throughout ART cycles. Some authors additionally propose that fertility clinics should adopt “opt-out” psychosocial models in which mental health assessment is routinely integrated into fertility care rather than offered only following overt psychiatric deterioration [12]. Psychologists and other mental health professionals represent central figures in the provision of infertility-related psychosocial care. Their role extends beyond the treatment of formal psychiatric disorders and includes screening, psychoeducation, supportive counseling, cognitive-behavioral interventions, couple-based interventions, coping enhancement, grief support, and emotional monitoring across treatment phases. The ESHRE psychosocial guideline differentiates between routine psychosocial care, infertility counseling, and psychotherapy. Routine psychosocial care may be delivered by all fertility staff members, whereas specialized counseling and psychotherapy should remain the responsibility of trained mental health professionals [73]. Psychologists may therefore provide both direct therapeutic interventions and consultative support for fertility teams. Mental health professionals may additionally contribute to the implementation of screening procedures aimed at identifying patients at increased risk for emotional maladjustment before IVF treatment. Screening tools such as SCREENIVF, FertiQoL, PHQ-9, GAD-7, and HADS may facilitate early identification of vulnerable patients requiring more structured psychosocial support [48]. To operationalize these screening metrics into clinical practice, a stratified stepped care framework can guide the triage process by channeling patients into distinct low-, moderate-, or high-risk intervention pathways, as schematically illustrated in Figure 2.

Beyond individual interventions, psychologists may also support couples experiencing communication difficulties, dyadic stress, intimacy problems, and treatment-related relational strain. The relational nature of infertility frequently requires interventions targeting emotional reciprocity, dyadic coping, and mutual emotional regulation rather than exclusively individual psychopathology. Importantly, psychologists may contribute to the development of phase-specific interventions adapted to different treatment phases, including anticipatory counseling before treatment, emotional support during ovarian stimulation and waiting periods, and grief-oriented interventions following unsuccessful cycles or pregnancy loss. Reproductive specialists and fertility clinic staff occupy a central position in the early identification and management of psychosocial burden during ART. Because fertility physicians, nurses, embryologists, and administrative personnel maintain continuous contact with patients throughout treatment, they are uniquely positioned to recognize signs of emotional distress, relational difficulties, maladaptive coping, or treatment fatigue. The ESHRE guideline specifically emphasizes that routine psychosocial care should be considered a responsibility of all fertility clinic staff rather than exclusively mental health professionals. Several dimensions of fertility care organization may substantially influence patients’ emotional adjustment. These include communication style, continuity of care, accessibility of information, involvement in decision-making, waiting times, clinic environment, and staff sensitivity to emotional vulnerability. Fertility specialists may therefore contribute to psychosocial well-being not only through medical treatment itself, but also through the manner in which treatment is delivered and communicated. Current integrated fertility care models additionally emphasize the importance of collaborative interdisciplinary approaches involving embedded mental health professionals within fertility clinics. Embedding psychologists or counselors directly into fertility teams may facilitate timely referrals, reduce stigma associated with mental health care, improve accessibility, and normalize psychosocial support as a routine component of treatment [74]. Despite increasing recognition of the importance of psychosocial care in infertility treatment, substantial barriers continue to limit its systematic integration into routine clinical practice. One of the most consistently reported barriers is the underutilization of mental health services among infertility patients, even in the presence of clinically significant emotional distress. Prospective studies demonstrate that although anxiety and depressive symptoms are highly prevalent during ART treatment, only a minority of patients receive mental health support or even information regarding available psychological services. Importantly, referral to mental health services often appears poorly targeted, with highly distressed patients not necessarily more likely to receive psychosocial support than less distressed individuals [9]. Multiple structural, organizational, and patient-related factors contribute to this implementation gap. Frequently reported barriers include stigma surrounding psychological care, financial costs, geographical distance from specialized services, time constraints, limited insurance coverage, insufficient awareness regarding available support, and shortages of trained fertility mental health professionals [12,13,14,15,16,17,18,19,20,21,22,23,24,25,26,27,28,29,30,31,32,33,34,35,36,37,38,39,40,41,42,43,44,45,46,47,48]. Feasibility studies nevertheless suggest that routine psychosocial screening may be successfully integrated into daily fertility practice. In a prospective implementation study evaluating SCREENIVF in routine IVF care, uptake rates remained stable around 78–80%, while most patients considered the screening process useful and acceptable. However, even among patients identified as psychologically at risk, help-seeking behavior remained limited, reinforcing the importance of improving accessibility and reducing practical barriers to care. Another important challenge concerns fragmentation between reproductive medicine and mental health services. Fertility treatment and psychological support are frequently delivered in separate settings with limited communication between providers, potentially reducing continuity and coordination of care. Contemporary integrated behavioral health models therefore increasingly advocate for embedding mental health professionals directly within fertility clinics in order to improve interdisciplinary collaboration and facilitate patient access to psychosocial support. Finally, substantial heterogeneity still exists regarding how psychosocial care is implemented across fertility centers internationally. Although professional societies increasingly recognize psychosocial care as an essential dimension of fertility treatment, standardized protocols for screening, referral pathways, interdisciplinary collaboration, and longitudinal psychological monitoring remain inconsistently implemented across clinical settings [73,74]. Economic evaluation should accompany future implementation studies. Psychosocial services generate direct costs related to screening, professional time, digital infrastructure, and referral pathways, but may also produce savings if they reduce unplanned contacts, decisional conflict, crisis presentations, or premature treatment discontinuation. Because dropout can reduce the cumulative opportunity to complete clinically indicated ART cycles, interventions that improve treatment continuity may have economic as well as psychological value. Nevertheless, current evidence is insufficient to conclude that integrated psychosocial care is cost-effective. Future studies should examine cost per clinically meaningful improvement in quality of life, cost per avoided discontinuation, healthcare utilization, patient out-of-pocket burden, and equity of access rather than relying exclusively on pregnancy-related endpoints.

## 8. Future Perspectives

Future research should prioritize prospective evaluation of clinically feasible stratification models based primarily on validated patient-reported outcomes, psychiatric history, reproductive experiences, couple functioning, treatment phase, and treatment burden. Studies should define referral thresholds a priori and assess feasibility, acceptability, false-positive and false-negative classifications, change over time, and equity across sex, gender, cultural, and socioeconomic groups. Predictive performance should be evaluated through calibration and discrimination analyses before any model is used to influence clinical decisions. Additional priorities include male-inclusive interventions, suicide-safety pathways, ethical governance of digital monitoring, implementation outcomes, and cost-effectiveness. Exploratory biological correlates of stress vulnerability may warrant investigation, but they should currently be regarded as research variables rather than clinically actionable psychosocial biomarkers [75,76,77,78,79,80].

## 9. Conclusions

Infertility and ART are associated with psychological, relational, and decisional challenges that vary across individuals, partners, treatment phases, and reproductive outcomes. The available evidence supports routine attention to emotional well-being as a component of patient-centered fertility care. Psychosocial interventions can improve anxiety, depressive symptoms, infertility-related distress, coping, couple functioning, and quality of life. They may also support informed decision-making and treatment continuity, although their independent effects on pregnancy and live-birth rates remain uncertain.

This review proposes a conceptual framework for stratified psychosocial care based on repeated screening, clinical assessment, phase-specific needs, and proportional intervention intensity. The low-, moderate-, and high-risk categories are pragmatic clinical groupings and should not be interpreted as a validated prediction algorithm. Screening results must be integrated with psychiatric history, functional impairment, reproductive experiences, couple dynamics, patient preferences, and direct clinical evaluation.

Effective implementation requires clearly defined referral pathways, an explicit safety protocol for suicidal ideation and acute psychiatric instability, inclusion of male-factor and couple-specific needs, and collaboration among reproductive physicians, nurses, psychologists, psychiatrists, urologists or andrologists, and other relevant professionals. Digital tools may expand access but require transparent governance, clinical oversight, crisis-response procedures, privacy protection, and assessment of algorithmic bias.

Future prospective studies should establish the feasibility, predictive validity, clinical utility, equity, and cost-effectiveness of stratified psychosocial pathways. Until such evidence is available, the principal aim of integrated psychosocial care should remain the improvement of patient well-being, safety, relational adjustment, and continuity of care rather than an assumed enhancement of reproductive outcomes.

## Figures and Tables

**Table 1:** Suggested Operational Use of Psychosocial Screening Instruments in ART Care.

Instrument or Assessment	Suggested Timing	Indicative Threshold or Trigger	Interpretation	Responsible Professional	Suggested Action	Reassessment
PHQ-9	Baseline; after cycle failure; when mood deterioration is suspected	5–9 mild; ≥10 clinically relevant; ≥15 moderately severe; any positive response to item 9 requires direct assessment	Depressive symptom severity; not a diagnosis	Nurse, psychologist, physician, or trained team member	≥10: clinical assessment or referral; ≥15: prioritized mental-health assessment; item 9 > 0: same-day suicide-risk assessment	At major treatment transitions or within 2–4 weeks when elevated
GAD-7	Baseline; stimulation; waiting period; after adverse outcome	5–9 mild; ≥10 clinically relevant; ≥15 severe	Anxiety symptom severity	Nurse, psychologist, physician, or trained team member	≥10: targeted clinical assessment; ≥15 or marked impairment: specialist referral	At next major treatment phase or within 2–4 weeks
HADS	Baseline and follow-up where routinely used	0–7 non-case; 8–10 possible case; ≥11 probable clinically significant symptoms on either subscale	Anxiety and depressive symptoms in medical settings	Trained fertility or mental-health professional	8–10: repeat or clinical interview; ≥11: referral according to severity and functioning	At cycle outcome or within 2–4 weeks
SCREENIVF	Before IVF or ICSI initiation	At risk when at least one validated subscale threshold is exceeded; use version- and language-specific scoring	Risk of emotional maladjustment following treatment	Psychologist or trained fertility-team member	Discuss risk profile and offer stepped psychosocial support	After unsuccessful outcome and before a subsequent cycle
FertiQoL	Baseline; after repeated failure; before discontinuation decisions	No universal diagnostic cut-off; interpret domain scores and clinically meaningful deterioration	Infertility-specific quality of life	Psychologist, nurse, or physician	Target impaired emotional, relational, social, or treatment domains	Every cycle when burden is increasing or every 2–3 cycles
Clinical interview	Whenever screening is positive or concern is raised	Suicidal ideation, severe hopelessness, psychosis, mania, severe substance misuse, domestic violence, marked functional impairment, or inability to provide informed consent	Determines urgency, diagnosis, and individualized risk	Psychologist or psychiatrist; emergency clinician when required	Immediate safety pathway or tailored intervention	

**Table 2:** Proposed Stepped Psychosocial Care Model in Assisted Reproductive Technology (ART).

Risk Profile	Main Characteristics	Suggested Interventions	Monitoring Intensity
Low Risk	Mild or transient emotional distress; preserved coping abilities; adequate relational and social support; no major psychiatric history; good treatment adherence	Psychoeducation; routine psychosocial screening; supportive counseling; stress management strategies; digital educational resources; peer support interventions	Routine phase-specific monitoring during ART treatment
Moderate Risk	Persistent anxiety or depressive symptoms; infertility-related distress; maladaptive coping strategies; relational strain; previous unsuccessful ART cycles; elevated uncertainty intolerance; reduced emotional adjustment	Structured psychological support; cognitive behavioral therapy (CBT); acceptance and commitment therapy (ACT); mindfulness-based interventions; couple-based interventions; targeted supportive psychotherapy	Regular longitudinal psychological follow-up across treatment phases
High Risk	Clinically significant psychiatric symptoms or disorders; severe emotional dysregulation; trauma history; suicidal ideation; major relational dysfunction; severe treatment fatigue; repeated treatment failures; marked psychosocial vulnerability	Multidisciplinary mental health care; psychiatric evaluation and treatment; individualized psychotherapy; pharmacological treatment when clinically indicated; intensive couple or family support; crisis intervention; integrated psychosocial care pathways	Intensive and continuous multidisciplinary monitoring throughout treatment

**Table 3:** Phase-Specific Psychosocial Needs and Responses Across the IVF Pathway.

Treatment Phase	Common Psychosocial Needs	Recommended Response
Pretreatment assessment	Prognostic uncertainty, unrealistic expectations, psychiatric history, previous losses, couple disagreement	Baseline screening; psychoeducation; expectation setting; individualized care plan; male and couple assessment
Ovarian stimulation and monitoring	Physical burden, hypervigilance, medication-related mood changes, occupational conflict	Brief check-in; symptom normalization; practical coping plan; rapid access to psychological review when symptoms escalate
Oocyte retrieval and sample provision	Procedural anxiety, pain concerns, male performance pressure, shame or collection difficulties	Procedure-focused preparation; analgesia and anxiety planning; private and supportive semen-collection pathway
Embryo transfer	Heightened hope, fear of failure, perceived responsibility for outcome	Realistic communication; avoidance of behavioral blame; brief coping guidance
Waiting period	Rumination, intolerance of uncertainty, reassurance seeking, symptom monitoring	Scheduled contact; uncertainty-focused CBT or ACT strategies; limits on unhelpful symptom checking; digital support with crisis escalation
Negative β-hCG or pregnancy loss	Acute grief, shame, hopelessness, couple asymmetry, suicide risk	Compassionate outcome communication; distress and safety assessment; grief-informed support; planned follow-up
Decision to repeat treatment	Treatment fatigue, decisional conflict, financial and relational burden	Shared decision-making; reassessment of psychological resources; couple consultation; updated care plan
Repeated failure	Cumulative grief, demoralization, relational strain, depressive symptoms	Structured psychotherapy; psychiatric assessment where indicated; decision counseling; discussion of alternative pathways
Treatment discontinuation	Identity transition, unresolved grief, loss of future narrative	Discontinuation counseling; meaning reconstruction; discussion of donor conception, adoption, or child-free living according to patient values

Abbreviations: ACT, Acceptance and Commitment Therapy; CBT, cognitive behavioral therapy; β-hCG, beta-human chorionic gonadotropin; IVF, in vitro fertilization.

## Data Availability

No new data were created.

## Abbreviations

The following abbreviations are used in this manuscript:

ART	Assisted Reproductive Technologies
IVF	In Vitro Fertilization
WHO	World Health Organization
MAR	Medically Assisted Reproduction
HPO	Hypothalamic–Pituitary–Ovarian
HPA	Hypothalamic–Pituitary–Adrenal
MHS	Mental Health Services
I-STRESS	Infertility-Related Stress Model
GnRH	Gonadotropin Releasing Hormone
ESHRE	European Society of Human Reproduction and Embryology
FertiQoL	Fertility Quality of Life Questionnaire
ASRM	American Society for Reproductive Medicine
HADS	Hospital Anxiety and Depression Scale
PHQ-9	Patient Health Questionnaire-9
GAD-7	Generalized Anxiety Disorder-7
ICSI	Intracytoplasmic Sperm Injection
FPI	Fertility Problem Inventory
FPS	Fertility Problem Stress Questionnaire
ICQ-I	Illness Cognitions Questionnaire Adapted for Infertility
CBT	Cognitive Behavioral Therapy
ACT	Acceptance and Commitment Therapy
